# Inconsistent Strategies to Mitigate the Effects of *Batrachochytrium salamandrivorans*, Europe

**DOI:** 10.3201/eid3207.251271

**Published:** 2026-07

**Authors:** Philipp Böning, Stefan Lötters, Vojtech Baláž, Wouter Beukema, Jaime Bosch, Salvador Carranza, Andrew A. Cunningham, Jesse Erens, Matthew C. Fisher, Trenton W.J. Garner, Maarten J. Gilbert, Florian Glaser, Christian Gortazar, Elena Grasselli, Matthew J. Gray, Daniel Fernández-Guiberteau, Thierry Kinet, Rok Kostanjšek, David Lastra González, Arnaud Laudelout, An Martel, Albert Martínez-Silvestre, Claude Miaud, Maciej Pabijan, Amadeus Plewnia, Doris Preininger, Riinu Rannap, Sebastiano Salvidio, Maximilian Schweinsberg, Annemarieke Spitzen-van der Sluijs, Raf Stassen, Ilias Strachinis, Barbora Thumsová, Jonas Virgo, Jiří Vojar, Michael Veith, Frank Pasmans

**Affiliations:** Trier University, Trier, Germany (P. Böning, S. Lötters, J. Erens, A. Plewnia, M. Veith); University of Veterinary Sciences Brno, Brno, Czech Republic (V. Baláž); Reptile, Amphibian & Fish Conservation Netherlands (RAVON), Nijmegen, the Netherlands (W. Beukema, M.J. Gilbert); Biodiversity Research Institute (IMIB), CSIC-University of Oviedo-Principality of Asturias, Mieres, Spain (J. Bosch); Institute of Evolutionary Biology (CSIC-UPF), Barcelona, Spain (S. Carranza); Institute of Zoology, Zoological Society of London, UK (A.A. Cunningham, T.W.J. Garner); Imperial College School of Public Health, London (M.C. Fisher); North-West University, Potchefstroom Campus, Potchefstroom, South Africa (T.W.J. Garner); Technisches Büro für Biologie, Absam, Austria (F. Glaser); SaBio Instituto de Investigación en Recursos Cinegéticos IREC (UCLM-CSIC-JCCM), Ciudad Real, Spain (C. Gortazar); University of Genova, Genova, Italy (E. Grasselli, S. Salvidio); University of Tennessee, Knoxville, Tennessee, USA (M.J. Gray); Centre de Recerca i Educació Ambiental de Calafell (CREAC-GRENP-Ajuntament de Calafell), Tarragona, Spain (D. Fernández-Guiberteau); Natagora, Namur, Belgium (T. Kinet, A. Laudelout); University of Ljubljana, Ljubljana, Slovenia (R. Kostanjšek); Czech University of Life Sciences Prague, Prague, Czech Republic (D.L. González, J. Vojar); University of the Ryukyus, Nishihara, Japan (D.L. González); Ghent University, Merelbeke, Belgium (A. Martel, A. Plewnia, F. Pasmans); Catalonian Reptiles and Amphibians Rescue Center (CRARC), Barcelona, Spain (A. Martínez-Silvestre); CNRS, EPHE-PSL University, Montpellier, France (C. Miaud); Jagiellonian University, Kraków, Poland (M. Pabijan); Vienna Zoo, Vienna, Austria (D. Preininger); University of Tartu, Tartu, Estonia (R. Rannap); University of Duisburg-Essen, Essen, Germany (M. Schweinsberg); Ministry of Defence, The Hague, the Netherlands (A. Spitzen-van der Sluijs); Biota.lu sàrl, Hostert, Luxembourg (R. Stassen); Aristotle University of Thessaloniki, Greece (I. Strachinis); Charles University, Prague (B. Thumsová); University of Bochum, Bochum, Germany (J. Virgo)

**Keywords:** fungi, animal health law, *Batrachochytrium salamandrivorans*, *Bsal*, chytridiomycosis, disease management, emerging infectious diseases, Europe

## Abstract

Emerging infectious diseases are one of the biggest challenges in a globalized world. To date, resources have been allocated to prevent and control the spread of zoonotic and livestock pathogens. We argue that, in line with the One Health approach, equitable efforts, financial resources, attention, and coordination are required for wildlife-only pathogens to halt biodiversity loss. Deploying the amphibian fungus *Batrachochytrium salamandrivorans* as a model, we demonstrate the unbalanced efforts among countries in Europe regarding surveillance, disease response, prevention, public outreach, and research. We compare investments with *B. salamandrivorans*–free countries such as the United States, concluding that structural resources are urgently needed to curb the effects of this fungus within Europe and beyond. We encourage dialogue among authorities, researchers, and stakeholders and propose a coordinated European Union–level program of €6–10 million over 5–7 years to implement *B. salamandrivorans* action plans and define structural funding requirements for future wildlife disease mitigation.

The effects of the Anthropocene on ecosystem, human, and animal health renders emerging infectious diseases (EIDs) key threats in the globally recognized One Health approach. Although some EIDs transmitted from wildlife affect human or domestic animal health, others drive wildlife declines only (*1*–*4*). Those wildlife-only EIDs can have far-reaching consequences for ecosystem functioning and human health (*4*–*7*). In detail, disease-driven alterations to community composition can have cascading effects across trophic levels, resulting in major downstream impacts on nutrient cycles and food web members (*1*,*8*–*12*). However, despite the overall definition and interpretation of One Health, wildlife-only EIDs are not given the same priority for mitigation as zoonotic or livestock EIDs (*13*,*14*). Conversely, approaches to consider and implement wildlife diseases into One Health do exist (*15*,*16*). In this article, we urge that those approaches be expanded to wildlife-only diseases and that the mitigation of disease threats to biodiversity be established as a key component of One Health strategies (*17*). Using an amphibian epidemic currently observed in Europe as an example, we demonstrate that structural resources are necessary to globally curb disease-driven loss of biodiversity and propose a coordinated European Union (EU)–level funding benchmark over >5 years to support this effort and guide dialogue among authorities, stakeholders, and researchers in Europe. Their effects on biodiversity demand that EIDs are approached using integrated and collaborative efforts between different authorities (i.e., in Europe: Environment and Health and Food Safety Departments of the European Commission) and should be properly addressed within One Health.

## Amphibian Chytrid Fungi

**Figure 1:**
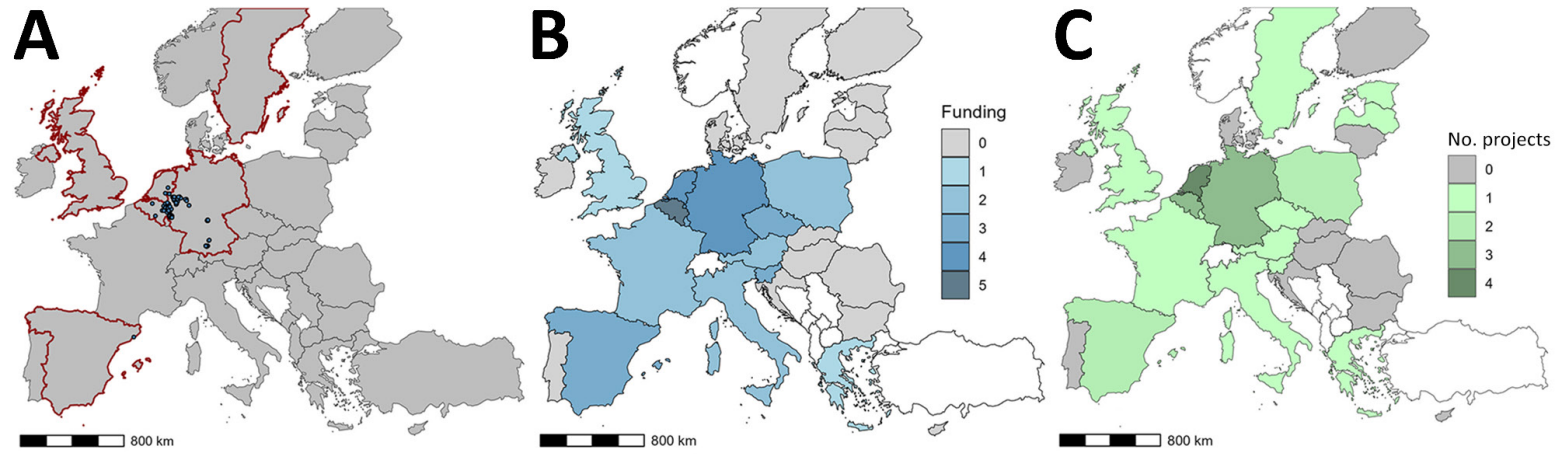
Efforts by country in the European Union and United Kingdom in study of inconsistent strategies to mitigate the effects of *Batrachochytrium salamandrivorans*, Europe. A) Known *B. salamandrivorans* sites in wild caudate populations (indicated by black dots) (*29*) and countries with *B. salamandrivorans* records from captive and wild populations (outlined in red) (Appendix 2 Table). B) Funding in Euros per country during 2013–2025 in classes (0 = no data; 1 = <50,000; 2 = 50,000–100,000; 3 = >100,000–500,000; 4 = >500,000–2 million; 5 = >2 million) C) Number of *B. salamandrivorans* projects per country during 2013–2025 in classes (0 = no data; 1 = 1–4; 2 = 5–9; 3 = 10–19; 4 = >20 projects).

The skin fungal disease chytridiomycosis, induced by *Batrachochytrium dendrobatidis*, has caused undeniable harm to global amphibian diversity, placing it among the top 5 threats to amphibians (*18*,*19*). Similarly, another batrachochytrid fungus, *Batrachochytrium salamandrivorans*, is emerging, potentially threatening salamander species (Caudata) and amphibian communities outside its native range (*20*–*24*) and possibly rising as a major species conservation challenge in the upcoming decade. *B. salamandrivorans*, originating from Asia and likely transported to Europe by the pet trade, was discovered in 2013 in the Netherlands and can cause lethal infection in most European salamander species (*20*). Its recent discovery and relatively limited observed distribution suggest *B. salamandrivorans* to be in a relatively early stage of invasion in Europe, which would make it a prime candidate for pathogen containment and elimination (*24*–*27*). However, after 2 decades of presence in Europe, >130 known outbreaks of *B. salamandrivorans* chytridiomycosis have occurred in the wild, affecting >6 native species in 4 countries in Europe (Figure 1, panel A) (*27**,**28*). Although natural *B. salamandrivorans* dispersal appears to be a relatively slow process with an estimated range expansion of 5–16 km/year (*27*), long-distance and likely human-mediated dispersal and independent introductions have been documented (*26*–*29*). In addition, anurans might serve as transmission and dispersal vectors, and *B. salamandrivorans* has been detected in captive amphibians (caudates and anurans) in private and zoo collections in 5 countries in Europe (Figure 1, panel A) (*30*). The pathogen is likely to be more widespread than currently recognized because it can be carried by some host species without causing disease, and when disease does occur, clinical signs are not pathognomonic (*29*).

## Current Situation in Europe

The EU initially responded to the novel threat of *B. salamandrivorans* by funding the development of the *Batrachochytrium salamandrivorans* Action Plan in 2017 (*24*). *B. salamandrivorans* has been listed in the European Animal Health Law (AHL) (*31*) since April 1, 2021. Of the 64 infectious diseases listed in the AHL, *B. salamandrivorans*–induced chytridiomycosis is the single wildlife-only EID that is subject to EU-wide regulations and the sole pathogen listed for biodiversity conservation reasons. The AHL listing of *B. salamandrivorans* in categories D and E dictates EU member states to conduct surveillance and prevent the spread of this pathogen. In an ideal scenario, such efforts would include both passive and active surveillance through early warning systems and targeted research, in addition to mitigation actions to curb *B. salamandrivorans* spread. Yet, current surveillance efforts are insufficient to detect *B. salamandrivorans* in its invasive range. The current observed distribution, the number of outbreaks, and the overall impact on species in Europe do not fit the predicted scenarios based on the potential host range and presumed unrestricted dispersal across most of Europe (*23**–**29*,*32**–**34*) (Figure 1, panel A). Therefore, the pathogen in Europe is still likely to be overlooked in the wild (*29*,*33*).

We compiled key information on *B. salamandrivorans* detection and funding until 2025 in 15 of 27 European Union countries and the United Kingdom since its discovery (Figure 1; Appendix 1 Table). We identified funding resources in 13 countries (12 EU countries and the UK) (Figure 1, panel B), as well as 16 countries with *B. salamandrivorans* projects (15 EU countries and the UK) (Figure 1, panel C; Appendix 2 Table). Response actions to *B. salamandrivorans* risk, either through funding or projects, are unknown in the other 12 countries, but are likely to be limited, if present at all. Among known actions, >60% of the tested sites and ≈71% of all samples tested for *B. salamandrivorans* presence (both in the wild and in captivity) were taken in the 4 countries with outbreaks in the wild (Belgium, Germany, the Netherlands, and Spain (Figure 1, panel A). Surveillance activities in natural environments and among wild amphibians occur 10 times more frequently than those targeting captive amphibians (46,578 vs. 4,940) (Appendix 1 Table), thereby limiting the capacity to detect a major transmission pathway of the disease. This discrepancy reveals either an immense Europewide sampling gap in captive amphibians, or, if testing is present but not reported, an unstructured, nontransparent reporting system for this pathogen. That deficit is further illustrated by publications with punctual data sampling (*28**,**29**,**33**–**40*).

In countries where *B. salamandrivorans* outbreaks have not been reported (N = 11) (Figure 1; Appendix 1 Table), only Austria and Luxembourg have sampling at a level commensurate with efforts expended in countries where the pathogen has been detected (Appendix 2 Figure). Regarding *B. salamandrivorans*–related funding across EU member states and the United Kingdom since the fungus’s scientific description in 2013, the overall volume reached >€12 million. So far, this money has been used for passive surveillance measures across and within member states through the establishment of a network in Europe consisting of 16 diagnostic centers and, for some countries, the development of national and regional action plans and risk analyses. These measures, combined with active measures, have led to the current knowledge on *B. salamandrivorans* in Europe as previously described. However, most (89%) of that funding was used by the 4 countries in which the fungus is present in the wild (Figure 1, panel A). In contrast, 7 other countries had access to funding of <€100,000 (Appendix 1 Table). Given those findings, *B. salamandrivorans* is likely still overlooked in Europe, and the current spatial distribution is skewed toward countries with access to funding.

**Figure 2:**
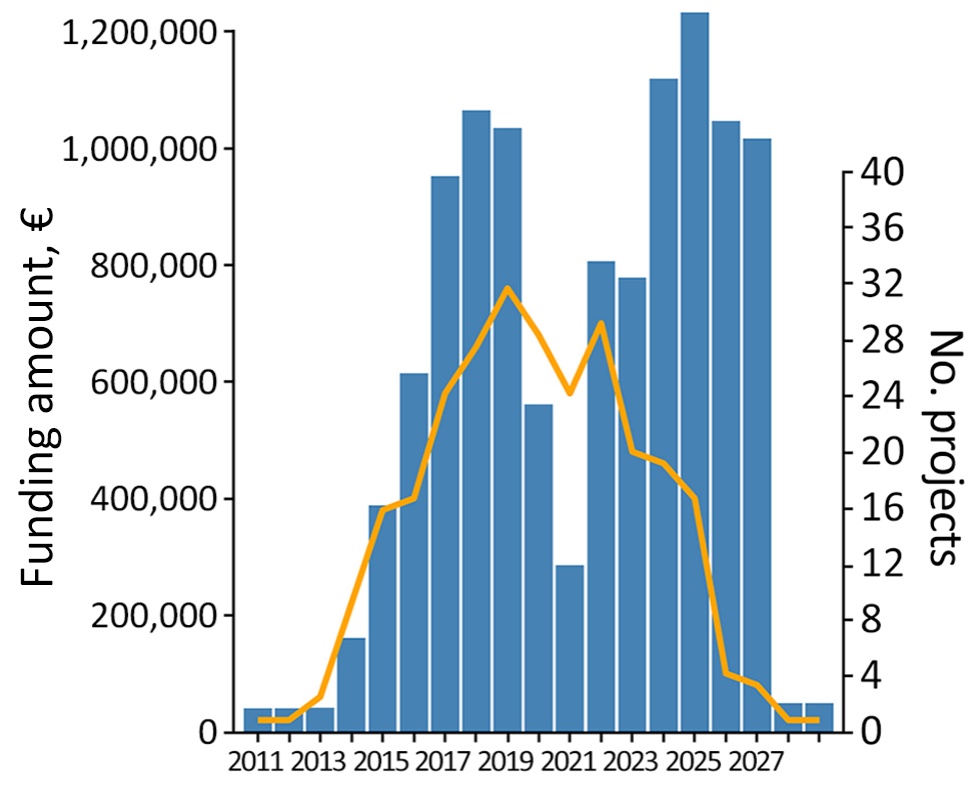
Overview of granted funding and projects per year in study of inconsistent strategies to mitigate the effects of *Batrachochytrium salamandrivorans*, Europe. Amount of funding per year is based on data in Appendix 2 Table. Yellow line indicates the number of projects per year. Multiyear projects were divided by their duration.

Overall, 77% of the projects identified were dedicated to *B. salamandrivorans* only. Of the 114 *B. salamandrivorans* projects identified, ≈83% aimed at *B. salamandrivorans* in the wild, of which ≈45% had funding for >3 years. In contrast, only 7% of the projects focused on the pathogen in captive facilities only. Moreover, projects differed in their components: 66.6% of projects included the detection of the fungus and 30% included its mitigation. For projects that incorporated conservation action as a project component, 43% of projects applied in situ conservation, whereas only 6% included ex situ conservation. A combination of in situ and ex situ conservation was used in 17.5% of all projects. None included rewilding actions (Appendix 1 Table). Overall, 38.6% of all projects focused additionally or exclusively on *B. salamandrivorans* fundamental research with a funding volume of ≈€9.8 million (80% of total money available) (Appendix 1 Table). That investment yielded fundamental knowledge of diversity, epidemiology, and disease ecology, resulting in the development of diagnostics and treatment strategies for captive urodeles. However, additional funding is needed for continued surveillance and development of sustainable, efficient, and long-term disease mitigation of natural populations (*24*–*27*,*29*–*35*,*41*). Since 2013, *B. salamandrivorans* funding experienced 2 peaks (≈€1.1 million in 2018 and ≈€1.2 million in 2025), explained by the number of projects that started and reached their midterm in 2019 and by the number that reached their midterm and end in 2025 (Figure 2; Appendix 1 Table). So far, available (fixed) funding for 2026 and 2027 is above ≈€1 million per year followed by 2 years with funding below €100,000; that funding is shared between 4 projects only (Figure 2; Appendix 1 Table). With regard to donors, 68% (≈€8 million) came from government funding, whereas ≈3% (€417,039) were granted by nongovernmental organizations and <1% (€101,887) by universities. The EU invested ≈27% (≈€3.3 million) (Appendix 1 Table).

## Comparisons and Need for Change

*B. salamandrivorans* sampling is insufficient in the EU for landscape-level detection and control. The limited-to-nonexistent enforcement of the AHL is exemplified by an almost complete lack of mitigation actions at known sites with infected wild populations, despite the availability of an action plan, including guidance on development of mitigation strategies (*24*,*25*). National *B. salamandrivorans* Action Plans exist in only 3 Member States (Belgium, Czech Republic, Luxembourg), but only Belgium and Luxembourg have incorporated the fungus into a (temporal) national strategy (Appendix 1 Table). Apart from that strategy, if actions are taken, they are mostly limited to raising public awareness and ex situ rescue of infected animals, often without a clear perspective of potential reintroduction and with uncertain effects on disease epidemiology. A few countries have invested in *B. salamandrivorans* research (Appendix 1 Table), but progress in both fundamental science and disease management is constrained by the paucity of studies testing the feasibility and effectiveness of mitigation or elimination strategies. Notable exceptions include a *B. salamandrivorans* outbreak in Catalonia, Spain, in 2018 that was met with rigorous actions that at least temporarily contained infection (*26*). The Netherlands has recently allocated €2 million to research on *B. salamandrivorans* disease ecology aiming to further develop in situ mitigation, and the EU recently invested €2.5 million (Horizon Europe/ERC101096163-GLOSSI) in fundamental research that might translate to novel *B. salamandrivorans* mitigation strategies in the future (e.g., long-term and in situ mitigation increasing host resistance and/or decreasing pathogen virulence). However, overall, the current, minimal efforts on a Europe-wide scale are unlikely to reduce further spread of *B. salamandrivorans* and prevent it from eventually reaching centers of caudate endemism in southern Europe. That probability is highlighted by the previous money flow and resulting projects conducted in those areas (Italy, Portugal, Spain, and the entire Balkan Peninsula), which did not surpass €400,000 and 2 projects per country (Figure 1; Appendix 2 Table).

Any serious attempt to control an EID requires sufficient resources, in this case at least those necessary to support effective surveillance and mitigation actions as required by the AHL. Certainly, the risk *B. salamandrivorans* poses to Europe’s unique amphibian biodiversity and its potential of inducing a trophic cascade (*42*) should urge investment in resources to develop sustainable mitigation strategies (*24**,**25*). That risk extends beyond Europe to other yet naive regions such as northern Africa and North and South America. Resources invested in Europe automatically lead not only to previously mentioned actions but simultaneously follow approaches like One Health and reduce risk in and spillover to nonaffected countries (*43*). The time is now to enhance resources against the pathogen; once EIDs become widely established, enormous efforts are required to slow down epidemics in the wild (*26*). Our concern is that this might also be the case for *B. salamandrivorans*. If the current known distribution is representative of the actual distribution, we might still be in a position to enact effective mitigation strategies. The shown inadequacy of monetary resources and the coupled lack of *B. salamandrivorans* control measures in the EU become more evident by comparing them with resources spent on controlling other AHL-listed pathogens with established surveillance and mitigation structures. For example, African swine fever (ASF), an emerging disease of both wildlife and livestock, receives broad attention by decision-makers. In this case, because the pathogens differ in their epidemiology, host range, and economic effects, ASF is not used as a biologic comparator but as a policy benchmark to illustrate the differences in political priorities, funding allocation, and how control measures are implemented under the same legislative framework. This level of attention has led to massive investments in elimination and education measures with estimated funds at ≈€215 million for 2014–2023 (*44*,*45*). In detail, those EU funds, through the European Food Safety Authority for example, supported scientific projects regarding disease risk assessments. In addition, they include the cofinancing of national surveillance plans by the European Commission, which include active surveillance activities such as culling, carcass disposal, or physical barriers to prevent disease spread and passive surveillance in domestic pigs and wild boars; population management of wild boars; and biosecurity measures (https://www.efsa.europa.eu/en/topics/topic/african-swine-fever#latest). However, during that period the EU invested only 0.9% (€900,000) of the ASF funding (≈€215 million) in *B. salamandrivorans* mitigation, clearly showing that the EU-wide responses established for ASF were not feasible for *B. salamandrivorans*. Despite some initiated activities in some countries on different administrative levels, those efforts remain far less structured and consistent than those activated for ASF.

Examining how the United States has dealt with the threat of *B. salamandrivorans*, where the pathogen is not yet known to occur, the need for the financial and structural efforts we advocate for Europe become even more apparent. The United States has invested >$2 million in surveillance for *B. salamandrivorans* in wild amphibian populations (>100,000 samples) and has invested >$7 million in fundamental research, including estimating susceptibility of native species, investigating and modeling possible transmission dynamics in amphibian communities, evaluating disease management strategies, and using decision science to establish a response framework if emergence occurs (*46**–**49*). In comparison, ≈€1.2 million was invested in the EU in total (of which ≈66% included direct funding for research); 11,743 samples were tested in *B. salamandrivorans*–naive EU countries by May 2025 (Appendix 1 Table).

The North American Bsal Task Force serves as the unifying entity for that continent, bringing together scientists and stakeholders to identify research and policy needs and advocate for funding (*49*). The United States does not have specific legislation, such as the European Health Law, to direct funding toward *B. salamandrivorans* surveillance and response. Instead, funding has primarily come from state natural resource agencies, the US Geological Survey, and private conservation organizations that recognize the threat of this pathogen to native biodiversity. In addition, the US National Science Foundation has awarded funding for fundamental research on *B. salamandrivorans* and the possible threat of pathogen invasion through the captive amphibian trade. The availability of government funding for *B. salamandrivorans* research in the future, however, is uncertain, because many science-based programs are being cut by the current US administration. Recently, the threat of *B. salamandrivorans* and other pathogens has gained the attention of the US amphibian pet trade industry. US businesses overwhelmingly support the healthy trade of amphibians, and US consumers are willing to pay 75% more for pet amphibians that are not infected with *B. salamandrivorans* or other pathogens (*50*). This interest and demand led to the recent creation of the nonprofit Healthy Trade Institute, Inc., which is launching a healthy trade certification program for pet amphibians.

Thus, the threat of *B. salamandrivorans* is recognized by both public and private sectors in the United States, and proactively investing resources is agreed upon as essential to reducing likelihood of emergence and responding effectively if the pathogen is detected. Of note, research and policy have been closely linked in the United States; this strategy has proven highly effective in lessening the import by trade of known, and suspected, amphibian vectors of *B. salamandrivorans* (*51*).

## Conclusions and Policy Implications

In conclusion, we highlighted the financial gaps existing between livestock (ASF) and wildlife-only (*B. salamandrivorans*) diseases and uncovered funding differences between the United States, a country naive to *B. salamandrivorans*, and the EU, in which a few countries have reported *B. salamandrivorans* outbreaks. We call for the EU to take responsibility in amphibian conservation by implementing and enforcing the Animal Health Law across EU member states and their administrative units regarding *B. salamandrivorans* control measures in the wild and in captive facilities. Control measures in captive facilities especially include implementing improved biosecurity in the amphibian trade (i.e., following clean trade strategies) through mandatory health certification for amphibians, which remains largely absent in most member states despite its potential for effectively limiting pathogen introduction and spread.

These steps would require structural funding to (at the very least) cover costs of monitoring and activities inscribed in *B. salamandrivorans* action plans, to limit the effect of the wildlife-only EID on biodiversity in Europe in line with the European biodiversity strategy 2030 (*52*). In addition, it would involve fostering exchange between agencies funding fundamental research and those who are responsible for conservation action. Establishing clear and complementary funding connections would help to ensure that science informs action without reducing research to a mere service function. Subsequently, such coordination translates scientific findings into effective biodiversity management and disease control policies on all administrative scales, as well as in areas with high salamander diversity (especially southern Europe) and to species highly susceptible but naive to *B. salamandrivorans*, as already analyzed in the *Batrachochytrium salamandrivorans* Action Plan in Europe (*24*). To achieve this goal, we urgently call for a dialogue between policymakers, researchers, and stakeholders discussing and defining funding, coordination and implementation strategies, and priorities. For this urgent dialogue, we propose a coordinated EU-level program in the range of €6–10 million over 5–7 years focusing on 5 major components (Table) as a realistic starting point, based on preventive investment levels in comparable jurisdictions. (The United States has invested approximately $9 million in preventive *B. salamandrivorans*surveillance and research despite the absence of confirmed cases.)

**Table:** Proposed European Union–level funding framework for coordinated prevention and response in analysis of inconsistent strategies to mitigate the effects of *Batrachochytrium salamandrivorans*, Europe

Funding component	Primary objective	Expected policy impact
International coordination	Enhancing information share and coordination	Harmonized cross-border response and coordination
Disease monitoring	Early disease detection and monitoring spread	Compliance with Animal Health Law (*31*)
Disease prevention	Reduce introduction and transboundary spread risk	Compliance with Animal Health Law (*31*)
Applied and fundamental research	Develop evidence-based and sustainable management tools	Availability of decision-making and mitigation tools
Outbreak response	Outbreak containment and reduced disease impact	Compliance with Habitats directive (*53*)

As we face the reality that the EU and its member states lack action against wildlife-only diseases such as chytridiomycosis, we urge more support in prevention measures such as campaigns to raise awareness, rapid-response systems, collaborative conservation actions, the implementation and maintenance of a *B. salamandrivorans* working group in Europe, and research in both hosts and pathogen following the efforts made in the United States to properly address the current disease emergency. Those actions are necessary and applicable to all known and yet unknown wildlife EIDs to elevate their recognition to that given to other wildlife diseases and to fulfill Europe’s obligation to One Health. In contrast, failing to address wildlife diseases risks undermining decades of conservation efforts and accelerating biodiversity loss across Europe. Because wildlife pathogens can spread rapidly across borders and ecosystems, coordinated prevention and surveillance are essential at the European level. Recognizing wildlife disease management as an integral component of One Health policy is critical to safeguarding biodiversity, ecosystem stability, and long-term environmental health.

Appendix 1 Additional data from study of inconsistent strategies to mitigate the effects of *Batrachochytrium salamandrivorans*, Europe

Appendix 2 Additional information about inconsistent strategies to mitigate the effects of *Batrachochytrium salamandrivorans*, Europe
