# Supplementary material for: Inconsistent Strategies to Mitigate the Effects of Batrachochytrium salamandrivorans, Europe
Source: Emerg Infect Dis. 2026 Jul;32(7):e251271. doi: 10.3201/eid3207.251271 (PMC13322446; doi:10.3201/eid3207.251271)
Supplement: Appendix 2 — Additional information about inconsistent strategies to mitigate the effects of Batrachochytrium salamandrivorans, Europe [file 25-1271-Techapp-s2.pdf]

Article DOI: https://doi.org/10.3201/eid3207.251271

EID cannot ensure accessibility for supplementary materials supplied by authors. Readers who have difficulty accessing supplementary content should contact the authors for assistance.

Inconsistent Strategies to Mitigate the Effects of *Batrachochytrium salamandrivorans*, Europe

Appendix 2

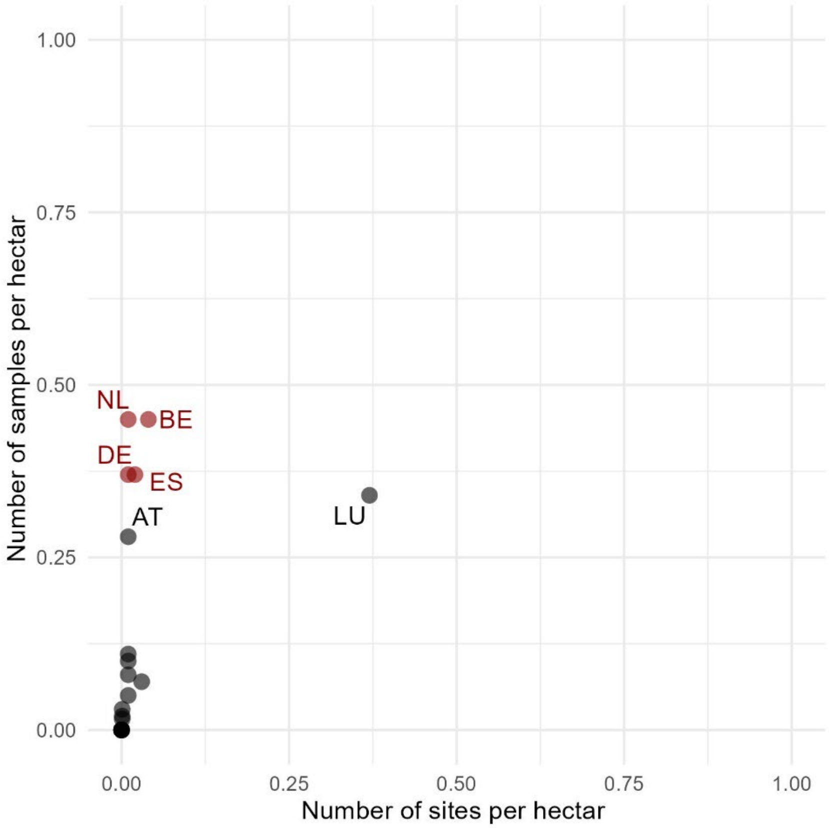

Appendix 2 Figure. Scatterplot of number of sample sites and number of samples per country per 10 km^2. Only countries with $\geq$0.25 samples per 10 km^2 are labeled (AT = Austria; BE = Belgium; CZ = Czech Republic; DE = Germany; ES = Spain; NL = Netherlands; UK = United Kingdom). Countries with *Bsal* records are highlighted in red. For raw data see Appendix 2 Table.

Appendix 2 Table. Raw data for Figure 1 in main article and Appendix 2 Figure. The table includes values for the amount of funding, funding category, country size (in square kilometers, after https://ec.europa.eu/eurostat/databrowser/view/reg_area3/default/table?lang = en&category = reg), number of sites (per hectare), number of specimens (per hectare), absolute numbers of sites, absolute number of specimens, number of projects, project category and information on *Bsal* presence in the wild (1) or absence (0) per country.

Code	Country	Funding	Country size	Funding category	Sites per $10km^2$	Specimens per $10km^2$	Sites	Specimens	Bsal	Projects	Project category
BE	Belgium	5250883	30667	5	0.04	0.46	134	1396	1	16	3
BG	Bulgaria	0	110996	0	0	0	0	0	0	0	0
CZ	Czechia	332687	78871	3	0.01	0.11	48	834	0	3	1
DK	Denmark	0	42925	0	0	0	0	0	0	0	0
DE	Germany	1790055	357569	4	0.01	0.36	504	12903	1	18	3
EE	Estonia	0	45336	0	0.01	0.05	39	243	0	1	1
IE	Ireland	0	69947	0	0	0	0	0	0	0	0
EL	Greece	4500	131694	1	0	0.02	17	225	0	1	1
ES	Spain	318016	505983	3	0.02	0.31	822	15572	1	9	2
FR	France	69000	638475	2	0	0.03	86	1900	0	3	1
HR	Croatia	0	56594	0	0	0	0	0	0	0	0
IT	Italy	58206	302073	2	0	0.01	26	400	0	4	1
CY	Cyprus	0	9253	0	0	0	0	0	0	0	0
LV	Latvia	0	64594	0	0	0	0	0	0	1	1
LT	Lithuania	0	65284	0	0	0	0	0	0	0	0
LU	Luxembourg	247236	2595	3	0.32	0.34	83	89	0	5	2
HU	Hungary	0	93012	0	0	0	0	0	0	0	0
MT	Malta	0	316	0	0	0	0	0	0	0	0
NL	The Netherlands	3651375.62	37391	4	0.01	0.37	29	1392	1	39	4
AT	Austria	89800	83882	2	0.01	0.28	70	2322	0	1	1
PL	Poland	97000	311928	1	0	0.04	138	1118	0	5	1
PT	Portugal	0	92226	0	0	0	0	0	0	0	0
RO	Romania	0	238398	0	0	0	0	0	0	0	0
SI	Slovenia	300000	20273	3	0.03	0.07	53	132	0	1	1
SK	Slovakia	0	49035	0	0	0	0	0	0	0	0
FI	Finland	0	338411	0	0	0	0	0	0	0	0
SE	Sweden	0	447424	0	0	0	0	0	0	1	1
UK	United Kingdom	42000	244423	1	0.01	0.1	210	2559	0	3	1
